# The Beneficial Effects of Physical Activity in Lung Cancer Prevention and/or Treatment

**DOI:** 10.3390/life12060782

**Published:** 2022-05-25

**Authors:** Gaetana Messina, Nicola Tartaglia, Antonio Ambrosi, Chiara Porro, Angelo Campanozzi, Anna Valenzano, Gaetano Corso, Alfonso Fiorelli, Rita Polito, Mario Santini, Marcellino Monda, Domenico Tafuri, Giovanni Messina, Antonietta Messina, Vincenzo Monda

**Affiliations:** 1 Department of Translational Medicine, Università degli Studi della Campania “Luigi Vanvitelli”, 80138 Naples, Italy; gaetana.messina@unicampania.it (G.M.); mario.santini@unicampania.it (M.S.); 2 Department of Medical and Surgical Sciences, University of Foggia, 71100 Foggia, Italy; nicola.tartaglia@unifg.it (N.T.); antonio.ambrosi@unifg.it (A.A.); angelo.campanozzi@unifg.it (A.C.); 3 Department of Clinical and Experimental Medicine, University of Foggia, 71122 Foggia, Italy; chiara.porro@unifg.it (C.P.); anna.valenzano@unifg.it (A.V.); gaetano.corso@unifg.it (G.C.); giovanni.messina@unifg.it (G.M.); 4 Department of Experimental Medicine, Section of Human Physiology and Unit of Dietetics and Sports Medicine, University of Campania “Luigi Vanvitelli”, 80138 Naples, Italy; marcellino.monda@unicampania.it (M.M.); antonietta.messina@unicampania.it (A.M.); vincenzo.monda@unicampania.it (V.M.); 5 Clinic of Child and Adolescent Neuropsychiatry, Department of Mental Health, Physical and Preventive Medicine, Università degli Studi della Campania, Luigi Vanvitelli, 81100 Naples, Italy; domenico.tafuri@uniparthenope.it

**Keywords:** lung cancer, healthy lifestyle, physical activity, oxidative stress, inflammation, lung cancer treatment

## Abstract

Lung cancer is the most lethal cancer: it has a significant incidence and low survival rates. Lifestyle has an important influence on cancer onset and its progression, indeed environmental factors and smoke are involved in cancer establishment, and in lung cancer. Physical activity is a determinant in inhibiting or slowing lung cancer. Certainly, the inflammation is a major factor responsible for lung cancer establishment. In this scenario, regular physical activity can induce anti-inflammatory effects, reducing ROS production and stimulating immune cell system activity. On lung function, physical activity improves lung muscle strength, FEV1 and forced vital capacity. In lung cancer patients, it reduces dyspnea, fatigue and pain. Data in the literature has shown the effects of physical activity both in in vivo and in vitro studies, reporting that its anti-inflammatory action is determinant in the onset of human diseases such as lung cancer. It has a beneficial effect not only in the prevention of lung cancer, but also on treatment and prognosis. For these reasons, it is retained as an adjuvant in lung cancer treatment both for the administration and prognosis of this type of cancer. The purpose of this review is to analyze the role of physical activity in lung cancer and to recommend regular physical activity and lifestyle changes to prevent or treat this pathology.

## 1. Introduction

There is a strong link between healthy lifestyle and pathologies such as cancer. Correct nutrition and regular physical activity can induce and/or reduce the molecular mechanism against cancer development and/or progression [1].

Lung cancer has long been the most common cancer in the world [2]. The processes leading to lung cancer establishment are different, and there are genetic factors and environmental factors such as lifestyle. Indeed, the obese and overweight conditions can increase the risk of cancer development through low-grade inflammation that represents a characteristic of these conditions.

For these reasons, a healthy lifestyle, in particular correct nutrition and physical activity, reduce and/or slow the inflammation cancer mechanism [3]. In this scenario, physical activity induces a molecular pathway which influences cancer risk and has important beneficial effects in the prevention of several types of cancer, lung cancer among them. It can reduce pro-inflammatory cytokines’ production and ROS production, and induce anti-inflammatory mediators’ production against cancer development [4]. It is known that physical activity acts on the immune response and that the characteristics of exercise influence immune and inflammatory processes [5]. Physical activity stimulates the immune system, improving the activity of macrophages and the secretion of anti-inflammatory mediators such IL-10 [6,7].

In addition, physical activity has an action both in cancer development and in its progression. It has effects on lung function, reducing the risk of respiratory diseases and cancer [3,4].

Exercise is a preventative factor and is important even after the diagnosis of cancer, improving the survival of subjects diagnosed and limiting mortality. In light of this evidence, the aim of this review is to clarify the role of physical activity in prevention and/or treatment of lung cancer and to elucidate the molecular mechanism undergoing the action of physical activity on lung cancer prevention and/or treatment.

## 2. Lung Cancer: Principal Information

### 2.1. Causes

Lung cancer is a leading cause of death, and there is no difference in the incidence according to sex. In addition, it has a high mortality rate as it is diagnosed for late stage [8]. During or after the treatment of one cancer, the patient may develop another one, including lung cancer [9]. The processes leading to neoplasia are various: genetic factors and lifestyle factors such as tobacco smoke, alcohol, nutrition, and physical activity which improve and/or slow the lung cancer establishment [10]. Regarding genetic factors, recent studies reported that the most important genes responsible for the development of lung cancer are EGFR, KRAS, MET, LKB1, BRAF, PIK3CA, ALK, RET, and ROS1 [11]. Mutations in the EGFR, KRAS and ERBB2 genes have been found in lung adenocarcinoma [12]. Non-small cell lung cancer (NSCLC) and small cell lung cancer (SCLC) is the best known. Being a silent tumor, lung cancer is very often fatal, because of its late diagnosis [13]. In terms of overall stage survival, it is 24 and 60 months and ranges from 97% and 92% for stage I patients, respectively, to 10% and 0% for stage IVB patients, respectively [14]. Although there have been new therapies and advances in the understanding of molecular mechanisms, lung cancer remains associated with physical, psychological, and social difficulties which exert a negative influence on the daily lives of patients [14]. While the extent of the disease is crucial among tumor-related factors, several host-related prognostic factors can decisively influence the scenario. Cigarette smoking promotes oncogenesis, disease progression and response to therapy [15]. Smoking is primarily responsible for lung cancer, inducing mutations in onco-suppressor genes, such as p53, and oncogenes, such as Kras [16,17]. is also able to influence the prognosis and the course of the disease as well as the outcome of therapy. In addition, a study on the impact of smoking on the prognosis of NSCLC has shown that quitting smoking within three months of the diagnosis of lung cancer has increased survival compared to those who continue to smoke [18,19]. The incidence of smoking is so high in the development of lung cancer is to be found in the chronic inflammation it causes, which is fatal for the development of oxygen radicals, cell damage leading to fatigue and deterioration of the individual [20].

### 2.2. Diagnosis

The symptoms of lung cancer patients vary widely. There are patients who arrive with asymptomatic diagnosis or symptoms such as cough, hemoptysis, intrathoracic spread, and distant metastases. About 10% of patients with lung cancer have paraneoplastic syndrome. Clubbing and hemostasis are characteristic of lung cancer [21], and loss of appetite, weight loss, fatigue, shortness of breath, chest or rib pain can be predictive of this type of cancer. Patients rarely present with only one symptom and the positive predictive value is higher when two or more symptoms are reported [21]. The presence of at least two of the above symptoms can give a predictive rate of about 10% [22]. Some lung cancers produce abnormally high blood levels of certain hormones or substances such as calcium. Lung cancer often produces metastases, which are controlled by many factors, including the interaction of various components in the lung cancer microenvironment, epithelial-mesenchymal transition transformation, and metastasis of cancer cells through blood vessels and lymphatics [5]. The molecular relationships are even more intricate. In addition, lung cancer leads to metastases in various organs and tissue, including to the brain [11]. Some genes act as a role in brain metastases of lung cancer, such as Twist2 and sparc/osteonectin, cwcv, and the SPOCK1 genes. Furthermore, osteolytic bone metastasis is more frequent in lung cancer [21,23]. The RANK/RANKL/OPG pathway plays an important role in bone metastases of lung cancer. Liver is also one of the most common sites of metastasis in patients with advanced lung cancer. Diverse cell populations in the liver immune microenvironment play a major role in tumor genesis and metastasis; indeed, the patients with lung cancer have the worst prognosis of liver metastases [23]. Once lung cancer begins to cause symptoms, it is usually visible on an X-ray or CT scan [21]. The diagnosis of lung cancer is generally confirmed with a lung biopsy or bronchoscopy. If the biopsy confirms lung cancer, your doctor will use other tests to determine the type of cancer and how far it has spread. Nearby lymph nodes can be tested for cancer cells with a procedure called mediastinoscopy, while imaging techniques such as CT scans, PET scans, bone scans, and an MRI or CT scan of the brain can detect cancer in other parts of the body [22].

### 2.3. Treatment

Treatment of lung cancer may involve resection of the tumor, but the decision is based on the type of lung cancer, how far it has spread and the functionality of the lungs. Many people with lung cancer, particularly smokers, have other lung or heart problems that make surgery difficult. Surgery is the preferred treatment for non-small cell lung cancer. In addition to surgery, treatment also involves radiation therapy and chemotherapy [23]. Fundamental is the use of combined chemotherapy, which is the use of more than one drug, often together with radiation therapy. Surgery is occasionally used, but only if the cancer is believed to be at a very early stage [24,25,26], which is rare. People whose tumors have metastasized or have spread to distant parts of the body are usually treated with chemotherapy or radiation therapy. Since metastatic lung cancer is very difficult to treat, the main objectives of treatment are to provide comfort and prolong life [27,28,29,30]. Current treatments can reduce tumors, which can reduce pain and other symptoms. Recent data also suggest that chemotherapy helps prevent the recurrence of lung cancer in patients with early stages of the disease [31].

## 3. Physical Activity: Summary Beneficial Effects

When we refer to physical activity, we mean any movement using skeletal muscles. In addition, we summarized physical activity in four principal subgroups:

○ Occupational
○ Household
○ Transport
○ Recreational or leisure-time

**Figure 1:**
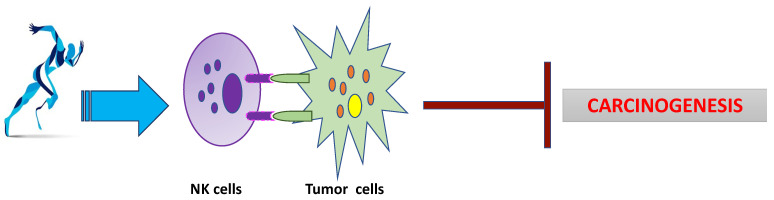
**The main biological effects of physical activity in tumor progression**: physical activity promotes the recognition of cancer cells by natural killers, preventing carcinogenesis.

Furthermore, physical activity is different with regard to intensity, strength, and time. To any physical activity we refer, it has many beneficial effects on body composition, insulin sensitivity, and blood glucose levels, and these effects have been demonstrated in multiple physiological and pathophysiological conditions [32]. From data literature, it is known that physical activity represents a real therapy for various pathologies, as it has beneficial effects on various organs and tissues and it can improve the state of health, preventing metabolic and inflammatory diseases, and acting on the central nervous system in terms of cognitive and psychological activity [33]. Physical exercise reduces the chronic inflammation and oxidative stress both in physiological and in pathophysiological states of the lung, such as lung cancer and/or cystic fibrosis, and it induces molecular mechanisms that act on anti-inflammatory mediators. In addition, it is involved in immune and metabolic functions, inducing physical and mental wellness, and it prevent many metabolic and inflammatory diseases such as cancer [34]. Physical activity can reduce the risk of the onset of these pathologies, and especially their severity, through different and complex molecular mechanisms. Moreover, the type of physical activity, intensity and duration are also able to modulate the immune and inflammatory response in a different way [35]. Indeed, it is well known that the prolonged secretion of pro-inflammatory cytokines can lead to the transformation of cells that eventually can lead to cancer. Exercise can reduce a variety of inflammation markers. Most premalignant cells are eliminated by innate immune cells in an immune surveillance process, particularly by natural killer (NK) cells specializing in the elimination of virus-infected cells or in the process of cell transformation. It has been reported that exercise leads to an increase in the number of circulating NK cells, as well as an enhancement of the cytotoxic function of NK cells [36]. In cases of chronic inflammation and sustained immunological pressure, a Darwinist micro-selection process takes place in which cancer cells acquiring further mutations become resistant to being killed by immune cells, surviving and making the emerging cancer less and less immunogenic. Eventually, the tumor can escape immune control, the growth rate accelerates, and the cancer becomes clinically detectable [37]. In this scenario, exercise can positively affect innate and adaptive immune responses after immune flight and subsequent tumor growth. The anti-inflammation induced by physical exercise has been demonstrated to involve a differential cytokine response represented by increased circulating IL-6 levels followed by a rise in IL-1ra and IL-10 levels and a suppression of TNF production. In addition, it could be protective against cancer by regulating the behavior of macrophages in the tumor microenvironment [38]. Research has shown that exercise exerts modulatory effects on macrophage metabolism, phagocytosis, chemotaxis, and anti-tumor activity [39,40,41] (Figure 1).

Focusing on the topic of the present review, physical activity is important in lung cancer prevention and in treatment and rehabilitation post-intervention.

## 4. Physical Activity in Lung Cancer Prevention

Exercise influences lung function and prevents the risk of infections and respiratory diseases. Data in the literature show that different mechanisms are involved in the relationship between cancer and physical activity through the regulation of chronic inflammation and the modulation of different substances acting as part of metabolic dysregulation such as insulin, glucose and sex hormones. In addition, it seems that physical activity has an impact on oxidative stress and immune function, modifying some crucial mechanisms related to the tumor microenvironment such as angiogenesis, proliferation, and apoptosis [42].

Much data in the literature has shown that there is a strong association between regular physical activity and low levels of inflammation.

**Figure 2:**
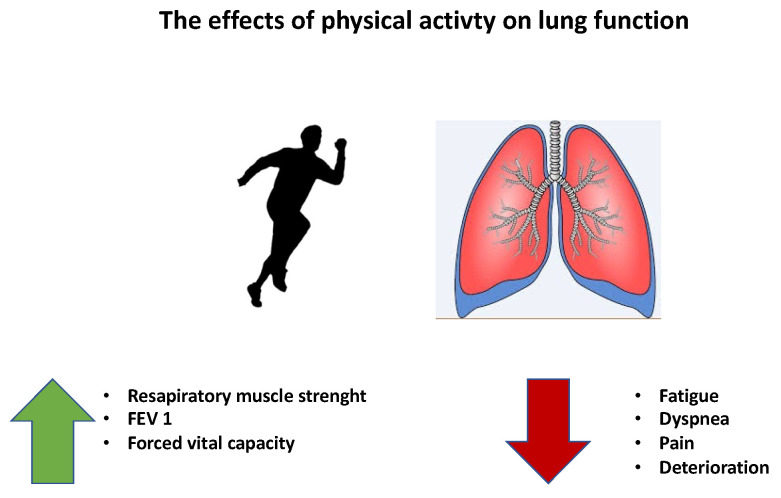
The principal effects of physical activity on lung function.

The anti-inflammatory response induced by regular physical activity is mediated by skeletal muscle contraction through the release of muscle-derived cytokines. Physical activity induces a marked increase in serum levels of cytokines involved in the regulation of inflammation, such as IL-10, the IL-1 receptor antagonist (IL-1ra), and IL-37 [43,44,45,46]. IL-6 is also capable of acting as an anti-inflammatory cytokine for several hours after exercise, as it reduces the production of pro-inflammatory cytokines in different tissues; this action is important against cancer development. Releasing IL-6 by muscle cells induced by physical activity leading to activation of important molecular pathways such as nuclear T cell factor (NFAT) and mitogenic protein kinase activated by glycogen-p38 (MAPK) [47]; therefore, the expression of TNFα or NF-kB increases during prolonged inflammatory responses [48]. In a physiological state, in the muscle of a subject who practices physical activity there is a decrease in pro-inflammatory macrophages of subtype 1 (M1) and an increase in anti-inflammatory macrophages of subtype 2 (M2). Furthermore, the production of PGC1α, which is an anti-inflammatory mediator, and which increases rapidly after a period of exercise, also generates the polarization of macrophages from pro-inflammatory M1 to anti-inflammatory [49]. Moreover, PGC1α is also able to suppress the expression of inflammation and increase the expression of anti-inflammatory cytokines, respectively [50]. In addition, physical activity is able also to modulate TLR expression on monocytes and macrophages; this modulation is influenced by sedentary lifestyle, inflammation status, and whether the disease has been consolidated. [51]. However, the molecule that could play a key role in mediating all of the positive anti-inflammatory effects induced by physical activity is the Mitogen Activated Protein Kinase (MAPK). This fuel-sensing enzyme, activated in contracting skeletal muscles, stimulates energy-generating pathways such as glycolysis and fatty acid oxidation, and decreases energy-consuming processes such as protein and lipid biosynthesis [52]. Physical activity mediated MAPK signaling accomplishes a dual purpose: it activates energy metabolism and can indirectly inhibit the inflammatory response induced by the NF-κB associated with chronic stress, likewise occurring in metabolic and inflammatory syndrome as well as in type 2 diabetes, obesity, and cancer [48,51]. The link between physical activity and cancer is also represented by adipose tissue. Indeed, physical activity, in general a healthy lifestyle, has a critical role in maintaining adipose tissue physiology by controlling adiposity deposition, the inflammatory state, immune responses and endocrine activity [53]. Furthermore, physical activity reduces the inflammation status of adipose tissue. The number of weekly bouts of exercise and their intensity are additional factors to consider when looking at the positive effects of physical activity on adipose tissue health. Exercise regulates the endogenous systemic environment by influencing cell processes and tumor growth [54], regulating glucose metabolism, immune and inflammatory factors, sex hormones, oxidative stress, and genomic instability [52,53,54] (Figure 2). In fact, hyperinsulinemia can contribute to the genesis of cancer, as binding to circulating IGF binding proteins and sex hormone binding globulins (SHBG) increases the bioavailability of IGF-1 and sex hormones, which may play a decisive role in cell proliferation. Physical activity can also reduce the risk of obesity-mediated cancer [53,54,55], since obesity is associated with an increased risk of developing up to 13 types of cancer through similar biological mechanisms [54]. Physical exercise, inducing weight loss, in postmenopausal women induces a reduction of estradiol and C-reactive proteins, reducing the risk of developing breast and endometrial cancer [50,55].

## 5. Physical Activity in Lung Cancer Treatment and Rehabilitation

Physical activity also has beneficial effects on some clinical symptoms, such as dyspnea, muscle atrophy, pain, fatigue, loss of appetite, and deterioration of physical fitness, all of which are attributable to the alteration of lung function due to lung cancer [54]. From the literature, we know that physical activity has numerous beneficial effects on lung function in inflammatory and genetic diseases such as sleep apnea nocturne and/or cystic fibrosis, ameliorating FEV1% and decreasing fatigue and improving quality of life (Figure 2).

### 5.1. Physical Activity in Lung Cancer Treatment

In lung cancer patients, physical activity is a non-pharmacological approach able to induce an amelioration in these patients In addition, it represents an adjuvant for chemotherapy and surgical treatment [56]. Exercise and physical activity can reduce inflammation and may induce molecular signaling pathways that support muscle mass building and stimulate beneficial metabolic adaptations [57]. Physical activity and exercise can be considered non-pharmacological interventions for the treatment of lung cancer that can improve fatigue, quality of life, lung function, muscle mass, strength and the psychological state, as demonstrated by several studies [58].

As for the action of exercise on muscle strength in people with lung cancer, there is not much literature on it [59]. Salhi et al. report that patients with lung cancer doing a 12-week rehabilitation program preserve muscle mass compared to patients with lung cancer who are sedentary [60]. From a clinical point of view, the effects of exercise in lung cancer patients are the improvement of fatigue and lung function and improved sleep quality, resulting in an improvement in the quality of life [58]. In patients with operable lung cancer, exercise prior to surgery reduces the risk of postoperative lung complications and improves rehabilitation [60]. High intensity preoperative exercise in fragile elderly patients undergoing pulmonary resection with lung cancer has improved rehabilitation both in hospital and at home [60,61,62], improving muscle strength and respiratory symptoms. In patients with non-operable lung cancer, exercise helps to maintain lung function and muscle strength [62].

### 5.2. Physical Activity in Lung Cancer Rehabilitation

Mounting evidence suggests that physical activity is safe in patients with lung cancer, both after surgery and during and after medical treatments. Studies report that all levels of training intensity are well tolerated by patients with lung cancer, and the frequency most often applied in different studies is two or three times a week, with the time per session ranging from 5 to 120 min. However, most patients with lung cancer are not quite sedentary, and several studies have reported low adherence and a high abandonment rate from physical activity programs [59,60,61]. Among the reasons for neglect, there are certainly the side effects from therapies and the disease itself, as well as a level of depression that occurs in these patients who lose interest and motivation. As for the biological and metabolic processes regulated by physical activity in lung cancer, the effect of physical activity on the immune system and therefore on the inflammatory response in different physio-physical conditions are not yet clear pathological is now evident, as is lung cancer [51]. Through physical activity, the cytokine response is improved, and physical activity, especially in the cellular microenvironment, induces a natural killer cell and macrophage response through the production of pro-inflammatory cytokines, which leads to the suppression of cancer cells [47]. In addition, exercise has an anti-angiogenic action, stimulating the release of VEGF in the muscle. In vivo and in vitro studies have reported that physical activity affects oncogenesis through the activation of apoptosis induced by p53. Studies in animal models of pulmonary adenocarcinoma have reported that mice subjected to physical activity compared to the control group had a significant reduction in primary tumor growth with a significant increase in intra tumor levels of p53 [61,62]. The beneficial effects of physical activity have been demonstrated both in vivo and in vitro, in fact the cell line of pulmonary adenocarcinoma (A549) treated with serum from subjects undergoing a physical activity program show a significant reduction in proliferation and survival compared to cells treated with serum from sedentary subjects or those treated with FBS only [48,63].

## 6. Conclusions

Regular physical activity is fundamental to reduce the risk of lung cancer development. For these reasons, it is considered as an adjunctive therapy to improve the management and reduce the poor prognosis of lung cancer. Physical activity should be introduced into the common practice of lung cancer treatment in combination with conventional therapies by creating guidelines for its use. In addition, performing a regular physical activity and reducing sedentary behaviors together with a reasoned selection of foods and dietary plans can influence the prognosis of the disease. Positive effects of those approaches likely pass through the influence of biological processes that are crucial in carcinogenesis, such as inflammation, immunity, and lung modulation.

## Data Availability

Data is contained within the article. Authors can use this data for research purposes only by citing our research article.
